# Sex Differences in Performance and Pacing Strategies During Sprint Skiing

**DOI:** 10.3389/fphys.2019.00295

**Published:** 2019-03-22

**Authors:** Erik Petrus Andersson, Andrew Govus, Oliver Michael Shannon, Kerry McGawley

**Affiliations:** ^1^Swedish Winter Sports Research Centre, Department of Health Sciences, Mid Sweden University, Östersund, Sweden; ^2^Department of Rehabilitation, Nutrition and Sport, La Trobe University, Melbourne, VIC, Australia; ^3^Human Nutrition Research Centre, Institute of Cellular Medicine, Newcastle University, Newcastle upon Tyne, United Kingdom

**Keywords:** cross-country skiing, elite athletes, head-to-head, metabolic demand, power output, time-trial

## Abstract

**Purpose:** This study aimed to compare performance and pacing strategies between elite male and female cross-country skiers during a sprint competition on snow using the skating technique.

**Methods:** Twenty male and 14 female skiers completed an individual time-trial prolog (TT) and three head-to-head races (quarter, semi, and final) on the same 1,572-m course, which was divided into flat, uphill and downhill sections. Section-specific speeds, choice of sub-technique (i.e., gear), cycle characteristics, heart rate and post-race blood lactate concentration were monitored. Power output was estimated for the different sections during the TT, while metabolic demand was estimated for two uphill camera sections and the final 50-m flat camera section.

**Results:** Average speed during the four races was ∼12.5% faster for males than females (*P* < 0.001), while speeds on the flat, uphill and downhill sections were ∼11, 18, and 9% faster for the males than females (all *P* < 0.001 for terrain, sex, and interaction). Differences in uphill TT speed between the sexes were associated with different sub-technique preferences, with males using a higher gear more frequently than females (*P* < 0.05). The estimated metabolic demand relative to maximal oxygen uptake ($\dot{V}$O_2max_) was similar for both sexes during the two uphill camera sections (∼129% of $\dot{V}$O_2max_) and for the final 50-m flat section (∼153% of $\dot{V}$O_2max_). Relative power output during the TT was 18% higher for males compared to females (*P* < 0.001) and was highly variable along the course for both sexes (coefficient of variation [CV] between sections 4–9 was 53%), while the same variation in heart rate was low (CV was ∼3%). The head-to-head races were ∼2.4% faster than the TT for both sexes and most race winners (61%) were positioned first already after 30 m of the race. No sex differences were observed during any of the races for heart rate or blood lactate concentration.

**Conclusion:** The average sex difference in sprint skiing performance was ∼12.5%, with varying differences for terrain-specific speeds. Moreover, females skied relatively slower uphill (at a lower gear) and thereby elicited more variation in their speed profiles compared to the males.

## Introduction

Cross-country sprint ski competitions are conducted over 3–4 h and involve an initial qualification time trial (a prolog) followed by three head-to-head knockout races (the quarter-finals, semi-finals, and final), with each race lasting ∼2–4 min (Andersson et al., 2016). Unlike other head-to-head endurance sports that require races to be completed successively on the same day (e.g., swimming, track athletics, or track cycling), the qualification race within a sprint skiing competition is conducted as a time trial, and therefore without direct contact with opponents. The subsequent head-to-head knockout races then involve six athletes competing together. A further distinction within cross-country sprint ski racing is the undulating terrain, which results in the use of several different sub-techniques within the two separate disciplines of classic and skate skiing (Stöggl et al., 2007; Andersson et al., 2010). These factors influence the athlete’s distribution of both power output and energetic resources during sprint skiing races (Andersson et al., 2016; Swarén and Eriksson, 2017), thereby creating unique challenges in terms of optimizing pacing strategies (Sundström et al., 2013; Andersson et al., 2016).

Swarén and Eriksson (2017) analyzed one female and one male cross-country skier and demonstrated highly variable speed and power output profiles during a sprint skiing time trial using the classic technique. The highest power outputs were generated at the beginning of steep uphills, while the downhill gliding sections involved no active generation of power output. Hence, sprint cross-country skiing is highly intermittent, involving periods of very high-intensity exercise interspersed with periods of lower-intensity exercise or recovery. This differs markedly from swimming, track running and track cycling events lasting ∼2–4 min, which are typically characterized by more even speed and/or power-output distributions (Corbett et al., 2009; Hanon and Thomas, 2011; Skorski et al., 2014). Instead, the intensity profile in cross-country skiing is more similar to the demands of cross-country mountain biking and road cycling over undulating terrain. In cycling, high power outputs are typical during uphills and power output profiles are intermittent over the course of a race, indicating a variable, terrain-specific pacing strategy (Jeukendrup et al., 2000; Abbiss et al., 2013). However, to date, there is no information relating to the power output distributions and simultaneous heart rate responses during a sprint skiing time trial on snow. Such information is likely important when planning training, preparing to race, and evaluating performance in cross-country skiing.

Previous studies have shown that performance and pacing strategies are different when athletes perform individually (i.e., during time-trial races) compared to when they come into direct contact with an opponent (i.e., during head-to-head races) (Corbett et al., 2012; Konings et al., 2016). For performance outdoors, this is at least partly due to the advantages associated with drafting, whereby power output and energy cost are substantially lower for the same speed when sheltered in the slipstream of an opponent (Bilodeau et al., 1994; Brisswalter and Hausswirth, 2008). Due to a reduced effect of drafting at lower speeds (Brisswalter and Hausswirth, 2008), it is logical to assume that a relative increase in effort and therefore speed is likely to occur during uphill sections of head-to-head races for any athlete attempting a breakaway. In addition, positioning and maximal speed capacities may be of crucial importance during knockout races in sprint skiing, especially in head-to-head situations when approaching the finishing straight. However, there is currently no information regarding differences in pacing strategies between a time trial and the head-to-head races in sprint cross-country skiing, or regarding positioning and performance during head-to-head sprint races.

In middle-distance track running, males appear to be ∼12% faster than females under race conditions (Coast et al., 2004; Cheuvront et al., 2005). In elite cross-country sprint skiers, Sandbakk et al. (2014) observed a sex difference in peak speed during an incremental treadmill test of 17% when using the skating technique. In contrast to track running, however, the characteristics of cross-country skiing are distinguished by several different sub-techniques involving upper- and lower-body muscle groups operating in various combinations. These different sub-techniques are referred to as “gears” (G) and the choice of gear is highly related to skiing speed, with slower skiers using lower gears during skating. The two main sub-techniques employed during uphill ski-skating are G2 and G3, where G2 is an asymmetrical sub-technique involving one poling action over every second leg stroke and G3 is a symmetrical sub-technique involving one poling action for each leg stroke (Andersson et al., 2010). Therefore, it is likely that the slower female skiers would use a higher percentage of G2 than G3 during uphill skiing and as a result, use the upper body to a lower relative extent than males (Kvamme et al., 2005). As well as on uphill terrain, G3 is also employed on level terrain. The G4 sub-technique is mainly applied on level terrain and slight downhills and involves one poling movement for two leg strokes. The action of skating without poling is referred to as G5 and is performed at high speeds on level and slight downhill terrain, with the body in a relatively crouched position to reduce air drag (Andersson et al., 2010).

Although studies evaluating cross-country skiing performance in the laboratory have revealed performance differences between males and females, no study appears to have compared performance and pacing strategies in a group of elite male and female skiers during a sprint skiing competition on snow. Thus, the aim of the current study was to describe elite cross-country skiers’ sprint skiing performance using an ecologically valid yet experimentally controlled approach. It was hypothesized that: (1) male skiers would perform significantly faster than female skiers and that this difference would be augmented during uphill skiing; (2) the skiers would utilize a variable terrain-specific pacing strategy, whereby the greatest power outputs would be attained on the uphill sections; (3) the average speed would be faster during the head-to-head races compared to the time-trial, with different speed profiles characterizing the two types of races.

## Materials and Methods

### Participants

Thirty-four elite cross-country skiers were recruited for this study, which was pre-approved by the Regional Ethical Review Board of Umeå University, Umeå, Sweden (#2016-443-31M) and conducted in accordance with the Declaration of Helsinki. All participants were informed of the nature of the study before providing written consent to participate. Three participants were aged <18 years, thus written consent was also provided by a parent or guardian. Of the 34 recruited skiers, 32 were members of the Swedish national senior and junior teams and two (both male) were previous members of the senior national team. Twenty of the skiers were male (age: 23.1 ± 4.4 years, height: 182.8 ± 6.9 cm, body mass: 75.7 ± 7.7 kg) and 14 were female (age: 21.4 ± 3.3 years, height: 171.3 ± 4.8 cm, body mass: 64.4 ± 5.2 kg). According to the International Ski Federation (FIS) ranking points system, the males and females had 86.9 ± 42.1 and 89.7 ± 53.1 sprint points, respectively, and 61.6 ± 30.9 and 77.3 ± 27.8 distance points (FIS, 2016). The FIS ranking system is based on a zero-point standard set by the top-ranked skier in the world, thus the best skiers have the lowest FIS points.

### Study Overview

The cross-country sprint skiing competition was organized by the Swedish Ski Association and was designed to simulate World Cup competition conditions. Unlike real-world sprint racing, all skiers completed all four races of the competition, i.e., the individual time-trial prolog (TT) and the head-to-head quarter-final (QF), semi-final (SF) and final (F). The race was 1,572 m for both males and females and involved the ski-skating technique. Skiers were instructed to warm up and compete exactly as they normally would during a regular sprint skiing competition. Rest periods between races simulated real-world racing conditions and the skier’s overall sprint performance was defined as time taken to complete all four races. Heart rate was recorded continuously from the beginning of the warm-up prior to the TT until the end of the cool-down after the full competition.

### Equipment and Measurements During the Preliminary Laboratory Test

Within 1 month prior to the study, maximal oxygen uptake ($\dot{V}$O_2max_) was determined using an uphill (7°), diagonal-stride, roller-skiing time-trial test on a treadmill (Rodby Innovation AB, Vänge, Sweden) over a distance of 700 and 800 m for females and males, respectively, as a part of their regular physiological monitoring (McGawley and Holmberg, 2014). The participants used Pro-Ski C2 roller skis (Sterners Specialfabrik AB, Dala-Järna, Sweden) equipped with the NNN (Rottefella AS, Klockarstua, Norway) binding system. The coefficient of rolling resistance was measured to 0.023. The participants used their own ski poles, which were equipped with carbide tips designed for treadmill skiing. For safety reasons, each skier wore a safety harness around their waist that was suspended from the ceiling and connected to an emergency brake. Participants controlled the speed of the treadmill by adjusting their position on the belt (Swarén et al., 2013). Respiratory variables were measured using an ergospirometry system (AMIS 2001 model C, Innovision AS, Odense, Denmark) and the $\dot{V}$O_2max_ during the time trial was based on the highest 30-s moving average. The $\dot{V}$O_2max_ and maximum heart rate during the time-trial was 72.7 ± 4.5 mL kg^-1^min^-1^ (5.5 ± 0.5 L min^-1^) and 188 ± 5 beats min^-1^, respectively, for the male skiers (*n* = 17), and 59.8 ± 3.7 mL kg^-1^⋅min^-1^ (3.9 ± 0.4 L min^-1^) and 188 ± 8 beats min^-1^ for the female skiers (*n* = 12).

### Equipment and Measurements During the Field Testing

#### Skiing Equipment, Blood Lactate, and Heart Rate

The skiers used their own racing skis, boots, and poles with a total mass of 3.2 ± 0.2 kg. The temperature of the snow was 0°C and the ambient temperature was 1.0 ± 0.1°C, calculated from three measurements taken ∼2 min before, approximately halfway through and ∼5 min after the competition. The dynamic friction coefficient between ski and snow was estimated to μ = 0.045, representing a typical value for a snow surface temperature of 0°C (Colbeck, 1994, unpublished data; Buhl et al., 2001). All skis were selected and prepared for prevailing snow conditions by a team of professional ski technicians using the same stone grind and glide-wax (Briko Maplus Med Base). The skis were glide tested over a 36-m downhill section four times for each ski pair by the ski technicians prior to and after the race. The glide time for all tested ski pairs (based on the average time for each pair and excluding the most deviating time) was 4.74 ± 0.04 s prior to the race and 4.80 ± 0.04 s after the race.

Participants’ height and body mass, as well as equipment mass, were measured in the morning prior to the sprint competition using an electronic measuring and weighing station (Seca 764, Hamburg, Germany). Blood samples (20 μl) were taken from the fingertip 1–3 min after each race and were analyzed for the determination of blood lactate concentration within 60 min of sampling (Biosen S-line, EKF diagnostic GmbH, Magdeburg, Germany). The Biosen system was calibrated with a standard solution of lactate (12 mmol L^-1^) prior to the analysis. The participants used their own heart rate monitors set at a sampling frequency of 1 Hz, including sports watches from Polar (M400, M600, and V800; Polar Electro Oy, Kempele, Finland), Garmin (645, 935, 735XT, 920 XT; Garmin, Ltd., Olathe, KS, United States) and Suunto (Spartan Sport Wrist HR Sports Watch; Suunto Oy, Vanda, Finland). Heart rate was expressed as a percent of the race-day peak value, which was calculated as the highest 1-s heart rate value measured during any of the races on the race-day. Missing heart rate data for two male and three female skiers, due to technical problems, resulted in available data for *n* = 18 males and *n* = 11 females.

#### Course Profile

**FIGURE 1:**
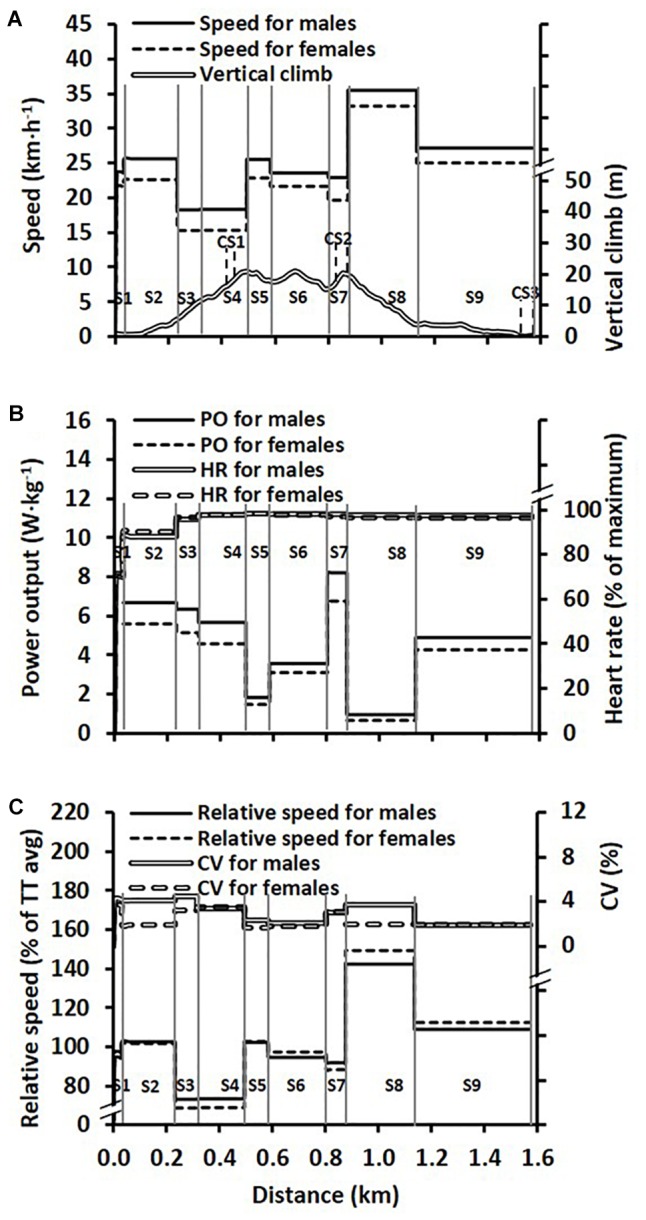
**(A)** Average skiing speeds over the different course sections (S1–S9) during the time trial (TT) race for male (*n* = 20) and female (*n* = 14) skiers, together with the course profile. The solid vertical lines represent the nine different sections and the dashed vertical lines represent the three camera sections (CS1–3). **(B)** Average relative power output (males: *n* = 20; and females: *n* = 14) over the different sections together with heart rate (HR) (females: *n* = 11, males: *n* = 18) during the TT. **(C)** Speed plotted as a percentage of the average (avg) TT speed over the different sections and the between-athlete coefficient of variation (CV) for this relative speed.

The sprint course included 56% of flat or undulating terrain, 22% of uphill terrain and 22% of downhill terrain divided into nine different sections (S1–S9), as illustrated in Figure 1A. The uphill and downhill sections were characterized by a minimum elevation difference of 5 m within the section, while sections with a total ascent or descent of <5 m were defined as flat or undulating. The course consisted of three uphill sections (S3 [83 m], S4 [178 m], and S7 [74 m]), with mean inclines of 3.9, 3.1, and 3.7°, respectively. There were two downhill sections (S5 [89 m] and S8 [259 m]), with mean inclines of -1.9 and -3.6°, respectively, and four flat sections (S1 [32 m], S2 [201 m], S6 [218 m], and S9 [437 m]) with mean inclines ranging between -0.7 and 1.4°. The maximal elevation difference of the course was 20 m with a total vertical climb of 29 m. The topography and distance of the course and the specific sections were measured 2 days prior to the race with a high accuracy Global Navigation Satellite System (GNSS) positioning equipment (Topcon HiPer II, Topcon Corporation, Tokyo, Japan). The system simultaneously receives signals from both the United States and Russian global navigation systems (GPS and GLONASS) and surveys positions at dual frequencies (L1/L2) in the real-time kinematic mode with 10 mm + 1 ppm and 15 mm + 1 ppm horizontal and vertical accuracies, respectively, as reported by the manufacturer. The section times were measured with the EMIT timing system (Emit AS, Oslo, Norway).

#### Video Recording, Sub-Techniques, and Cycle Characteristics

Three camera sections (CS1-3; Figure 1A) were included in the current study for the kinematical analysis of G2, G3, and G4 using Kinovea open source video-analysis software^1^. The total distances for the camera sections were 25, 35, and 40 m, with average inclines of 4.2, 3.7, and 0.3° for CS1, CS2, and CS3, respectively. All camera sections were marked with orange reference poles placed at 5-m intervals. The skiers were video recorded at a high resolution (1920 × 1080 progressive scan) in the sagittal plane using JVC GZ-R435BEU video cameras (JVC, corp., Yokohama, Japan) set at 50 Hz, with a shutter speed of 1/500^th^ of a second. The video cameras were positioned on fixed tripods placed perpendicular to the track at a distance allowing coverage of the entire sections. Due to an error detected in the orientation of the camera positioned in CS2, the distance skied using specific gears could not be determined accurately and therefore no cycle lengths were reported for this camera section.

All four main ski-skating gears were used in the current study. However, the first 30-m section (i.e., S1) was performed using the classic double-poling sub-technique in all races and by all athletes, as is standard in a sprint race. The time and number of movement cycles for the specific gears used in CS1-3 were analyzed from the video. One movement cycle was defined as a complete left and right leg stroke, together with the accompanying poling action (which differs for the different gears). For the calculation of cycle rate, the number of movement cycles was divided by the time spent using the specific gear. Cycle length was calculated by dividing the distance using a specific gear by the number of movement cycles. However, cycle length was not calculated if more than one gear was used in the specific camera section. Distance and speed for the specific gears were determined from the video footage using the reference poles.

#### Race Procedures

The sprint ski competition was performed in conjunction with a pre-season training camp at an international-standard ski stadium situated at an altitude of 710 m. The participants were asked to perform no more than light-intensity exercise (≤70% of maximal heart rate) the day before the race and to refrain from ingesting alcohol for at least 24 h. All skiers ate breakfast from a buffet stocked with familiar foods ∼3 h before the start of the sprint competition, and were instructed to take on foods and fluids throughout the competition as they normally would. Prior to the competition, carbohydrate intake was 1.6 ± 0.5 g/kg body mass for both the males and the females. During the competition, males consumed 1.0 ± 0.5 g of carbohydrate per kg body mass and females consumed 1.3 ± 0.3 g of carbohydrate per kg body mass. A thorough description of the nutritional intakes of these athletes recorded before and during the competition are available in a related article (Carr et al., 2018).

The race course was covered with a solid base (40–50 cm) of artificial snow that was covered by ∼5 cm of fresh, packed natural snow. The course was machine-prepared on the evening prior to the competition and once more on the morning of the competition, due to overnight snowfall, in order to optimize racing conditions. Unlike an official sprint competition, all skiers completed all four races, regardless of their result in each race, with rest periods between races simulating real-world racing conditions. The females competed first in each round, followed by the males, as is typical of real-world sprint ski racing. The TT started at 09:30 in the morning and was followed by the three head-to-head races (the QF, SF, and F). The times from start-to-start for all skiers were 103 ± 9, 34 ± 7, and 20 ± 3 min for the TT to QF, QF to SF, and SF to F, respectively.

The starting order for the TT was based on the FIS-point sprint ranking system for males and females, respectively, where the athlete with the best rank started first and a time gap of 15 s separated each skier. The head-to-head races were then performed with five males or seven females in each heat, resulting in four heats (A–D) per race for the males and two heats (A and B) per race for the females. Hence, 18 head-to-head heats were performed in total. The progress of skiers to the specific QF heats was based on performance during the TT, such that the fastest skiers in the TT were placed in heat A and the next fastest in heat B (and so forth down to heat D for the males). The two best skiers in each of the QF and SF heats were moved up one heat so that they started in a better heat during the SF and F, while the two slowest skiers were moved down one heat. This structure enhanced competitiveness, providing incentive to the athletes to finish in the top two of any heat and allowing those of relatively similar performance levels to race head-to-head in the SF and F.

### Calculations

The estimated average propulsive power output over the specific course sections was calculated as the sum of power exerted to elevate system mass (*m_sys_*; body mass together with equipment mass) against gravity (*g*), and to overcome snow friction (μ*_F_*) and air drag:

(1) $$Power\;output\;[w]=m_{sys}g\;\sin(\alpha )\nu +\mu_{F}m_{sys}g\;\cos(\alpha )\nu +0.5\rho C_{d}A\nu^{3}$$

where *α* is the average incline (°) of the course section, *v* is speed (m/s) and the final term describes the power from air drag acting on the skier, where ρ is the air density and *C_d_A* is the effective drag area (Swarén and Eriksson, 2017). Air density (ρ) was calculated from ambient temperature measured at the race location and air pressure obtained from an automatic weather station (HydroMet MAWS201M, Vaisala Oy, Helsinki, Finland) by dividing air pressure by the product of the specific gas constant and the ambient temperature. The effective drag area of the skier (*C_d_A*) was determined using a scaling function based on a reference value for an 80.2 kg skier with a *C_d_A* of 0.67 m^2^ (*C_d_A_ref_*) during uphill G3 skiing (Sundström et al., 2013; Ainegren and Jonsson, 2018):

(2) $$C_{d}A=C_{d}A_{ref}\times {\left(BM/BM_{ref}\right)}^{2/3}$$

where *BM* is the body mass of the participating skier and *BM_ref_* is the body mass of the reference skier. For downhill skiing, which mainly encompassed active ski-skating without poling in a tucked position (G5), a *C_d_A_ref_* of 0.34 m^2^ was used, and for flat terrain, a *C_d_A_ref_* of 0.63 m^2^ was used (Ainegren and Jonsson, 2018).

Since only average speeds were calculated for each section, the power output for acceleration (i.e., the rate of change of kinetic energy) was neglected and Equation (1) is thus a simplified version from that suggested by van Ingen Schenau and Cavanagh (1990) and used by Swarén and Eriksson (2017). As such, power output was not estimated for the first section (i.e., the 30-m double-poling section), where acceleration of the *m_sys_* compose a major part of the total power output. The average relative power output for uphill, flat and downhill terrain, as well as for the total TT race, was calculated as the sum of work for specific sections divided by the total time to cover these sections divided by the *m_sys_*. Average relative power output on the video sections was based on average speed obtained from the EMIT timing system data and the average incline derived from the GNSS measurements.

The average relative metabolic rate for the video sections was calculated by dividing the relative power output with an estimated average gross efficiency. The gross efficiencies for uphill skiing with G2 and G3 were fixed at 17.5 and 16.9% for CS1 and CS2, respectively, and for flat skiing with G3 and G4 at 15.8% for CS3. These values were used for both males and females and were based on previous results at approximately similar inclines (Losnegard et al., 2012; Sandbakk et al., 2013), as well as unpublished data from our laboratory. The $\dot{V}$O_2_ demand in mL⋅kg^-1^⋅min^-1^ was estimated by converting the metabolic rate in J⋅s^-1^ to J⋅min^-1^ and dividing by an energetic equivalent of 20.92 J mL^-1^ O_2_ (assuming 100% carbohydrate utilization) and the skier’s *m_sys_*.

### Statistics

The Statistical Package for the Social Sciences (SPSS 21, IBM, Corp., Armonk, NY, United States) was used to carry out statistical analyses and the level of significance was set at α ≤ 0.05. Data were confirmed to be normally distributed by visual inspection of Q–Q plots and histograms together with the Shapiro-Wilks analysis and are presented as mean ± standard deviation (SD) or as median and interquartile range for cycle characteristic data with *n* ≤ 4. A mixed-model ANOVA was used to analyze skiing performance between males and females over (1) all the different course sections and (2) the three different terrain types. Within-athlete coefficient of variation of skiing speed across sections was calculated for the TT. In addition, within-athlete coefficient of variation of relative power output and relative heart rate during the TT was calculated for course sections 2–9. The between-athlete coefficient of variation in TT speed for uphill, flat and downhill terrain was also calculated. Bonferroni α corrections were applied to all ANOVA tests. If sphericity was violated, the Greenhouse–Geisser or Huynh-Feldt corrections were utilized for epsilon ≤ 0.75 or > 0.75, respectively. Partial eta squared effect size ($\eta_{p}^{2}$) was also reported for the ANOVA tests. Independent sample *t*-tests were employed to compare performance, metabolic demand and cycle characteristics between males and females and a dependent sample *t*-test was used to compare TT speed with the average head-to-head race speed. To compare the preference of sub-technique between males and females a Fisher’s exact test was used to analyze the frequencies of G2 and G2 mixed with G3 versus solely G3 in CS1-2, as well as G3 and G3 mixed with G4 versus solely G4 in CS3. The standardized mean difference (Cohen’s *d*, effect size) was computed as the mean difference divided by the pooled SD. Cohen’s *d* effect size was presented together with 95% confidence intervals for the *t*-tests. Tactical positioning during all head-to-head sprint races was determined by calculating Kendall’s Tau-*b* correlations between intermediate rankings on each section along the course and the final ranking, presented as mean and 95% confidence interval.

## Results

### Performance and Pacing During the TT

The 1,572 m TT was performed in 227 ± 11 and 254 ± 10 s for males and females, respectively (*P* < 0.001, Cohen’s *d* effect size [ES] = -2.7 [-3.5 to -1.7]). Relative time distributions for males and females, respectively, were 27.9 ± 0.8 and 29.6 ± 0.7% on uphill terrain, 54.9 ± 0.4 and 53.8 ± 0.6% on flat terrain, and 17.2 ± 0.5 and 16.6 ± 0.2% on downhill terrain. A main effect was observed for relative time spent on each form of terrain (*F*_1,38_ = 23040, *P* < 0.001, $\eta_{p}^{2}$ = 1.00), but not for sex (*F*_1,32_ = 0, *P* > 0.999, $\eta_{p}^{2}$ = 0.00), while there was an interaction effect between terrain and sex (*F*_1,38_ = 36, *P* < 0.001, $\eta_{p}^{2}$ = 0.53). Average relative power output during the TT was 3.7 ± 0.3 and 3.2 ± 0.2 W/kg for males and females, respectively (*P* < 0.001, ES = 2.5 [1.6–3.3]).

Average and relative speed, relative power output and heart rate across the nine sections of the TT are illustrated in Figure 1 for the males and females separately. For speed (Figure 1A), main effects were observed for section (*F*_4,121_ = 1873, *P* < 0.001, $\eta_{p}^{2}$ = 0.98) and sex (*F*_1,32_ = 65, *P* < 0.001, $\eta_{p}^{2}$ = 0.67), as well as an interaction effect between section and sex (*F*_4,121_ = 4, *P* = 0.004, $\eta_{p}^{2}$ = 0.12). Similarly, for relative power output (Figure 1B) main effects were observed for section (*F*_3,93_ = 2010, *P* < 0.001, $\eta_{p}^{2}$ = 0.98) and sex (*F*_1,32_ = 55, *P* < 0.001, $\eta_{p}^{2}$ = 0.63), as well as an interaction effect between section and sex (*F*_3,93_ = 20, *P* < 0.001, $\eta_{p}^{2}$ = 0.39). By contrast, for heart rate (Figure 1B) there was a main effect for section (*F*_2,45_ = 506, *P* < 0.001, $\eta_{p}^{2}$ = 0.95) but not for sex (*F*_1,27_ = 1, *P* = 0.42, $\eta_{p}^{2}$ = 0.02) and there was no interaction effect (*F*_2,45_ = 2, *P* = 0.17, $\eta_{p}^{2}$ = 0.07). For speed expressed as a percentage of the skier’s average TT speed (Figure 1C), main effects were observed for section (*F*_4,124_ = 2055, *P* < 0.001, $\eta_{p}^{2}$ = 0.99) and sex (*F*_1,32_ = 5, *P* = 0.04, $\eta_{p}^{2}$ = 0.13), as well as an interaction effect (*F*_4,124_ = 20, *P* < 0.001, $\eta_{p}^{2}$ = 0.39). The within-athlete coefficient of variation in speed between sections was 20 ± 2% for males and 24 ± 1% for females (*P* < 0.001, ES = 2.6 [1.7–3.4]). For power output between sections 4–9 the within-athlete coefficient of variation was 53 ± 2% for males and 53 ± 1% for females (*P* = 0.963, ES = 0 [-0.7–0.7]), while the same variation in heart rate was 4 ± 1% for males and 2 ± 1% for females (*P* = 0.008, ES = 1.2 [0.4–2.0]).

Relative to the average TT speed, uphill, flat and downhill speeds were 77 ± 2, 103 ± 1, and 129 ± 4% for males and 72 ± 2, 105 ± 1, and 133 ± 2% for females. There were main effects for terrain (*F*_1,39_ = 4152, *P* < 0.001, $\eta_{p}^{2}$ = 0.99) and sex (*F*_1,32_ = 11, *P* = 0.002, $\eta_{p}^{2}$ = 0.26) and there was an interaction effect between terrain and sex (*F*_1,39_ = 27, *P* < 0.001, $\eta_{p}^{2}$ = 0.46). The between-athlete coefficient of variation in speed on uphill, flat and downhill terrain was 6.9, 4.0, and 3.5% for the male skiers and 5.6, 3.3, and 3.2% for the female skiers. Relative power output on the uphill, flat and downhill terrain was estimated to 4.9 ± 0.4, 4.0 ± 0.3 and 1.2 ± 0.2 W/kg for males and 4.0 ± 0.3, 3.4 ± 0.2, and 0.9 ± 0.1 W/kg for females (significant main effects for terrain [*F*_1,39_ = 2639, *P* < 0.001, $\eta_{p}^{2}$ = 0.99] and sex [*F*_1,32_ = 54, *P* < 0.001, $\eta_{p}^{2}$ = 0.63], as well as an interaction effect [*F*_1,39_ = 25, *P* < 0.001, $\eta_{p}^{2}$ = 0.44]).

### Gear Choice and Kinematics During the TT

Performance, estimated physiological responses, choice of sub-technique (i.e., gear) and cycle characteristics on the two uphill camera sections during the TT are shown in Table 1. On the first uphill video section (CS1) the males were 20% faster than the females, generating a 23% higher relative power output and metabolic rate. On CS1, 19 of the male skiers (95%) and three of the female skiers (21%) used G3 exclusively, and the remaining skiers (5% of the males and 79% of the females) used G2 exclusively or a combination of G2 and G3. On CS2 the males were again 20% faster than the females, generating a 23% higher relative power output and metabolic rate. Here, eight of the male skiers (40%) and none of the female skiers used G3 exclusively, while the remaining skiers (60% of the males and 100% of the females) used G2 exclusively or a combination of G2 and G3. Table 2 shows the performance, estimated physiological responses, choice of gear and cycle characteristics for CS3 (i.e., the last 50-m of the TT), which was flat. Here the males were 14% faster than the females, eliciting a 23% higher relative power output and metabolic rate and skied with a 17% longer cycle length in G3, with no significant difference in cycle rate between the sexes.

**Table 1:** Performance, power output, estimated physiological response, choice of sub-technique (i.e., gear) and cycle characteristics for the two uphill camera sections (CS1 and CS2) during the 1,572 m sprint time trial.

	Uphill (4.2°) CS1			Uphill (3.7°) CS2		
		
Speed, power and metabolic demand	Males	Females	ES	Males	Females	ES
Speed (km⋅h^-1^)	15.9 ± 1.1^∗^	13.2 ± 0.9	2.6 (1.6–3.4)	16.8 ± 1.3^∗^	14.1 ± 1.0	2.3 (1.4–3.2)
Total power output (W⋅kg^-1^)	5.6 ± 0.5^∗^	4.5 ± 0.3	2.5 (1.6–3.4)	5.6 ± 0.5^∗^	4.5 ± 0.4	2.3 (1.4–3.1)
Power output against gravity (W⋅kg^-1^)	3.2 ± 0.2^∗^	2.6 ± 0.2	2.6 (1.6–3.4)	3.0 ± 0.2^∗^	2.5 ± 0.2	2.3 (1.4–3.2)
Power output against snow friction (W⋅kg^-1^)	1.9 ± 0.1^∗^	1.6 ± 0.1	2.6 (1.6–3.4)	2.1 ± 0.2^∗^	1.7 ± 0.1	2.3 (1.4–3.2)
Power output against air drag (W⋅kg^-1^)	0.5 ± 0.1^∗^	0.3 ± 0.1	2.4 (1.4–3.2)	0.5 ± 0.1^∗^	0.3 ± 0.1	2.1 (1.2–2.9)
Metabolic rate (W⋅kg^-1^)	31.9 ± 2.6^∗^	26.0 ± 1.9	2.5 (1.6–3.4)	33.0 ± 3.0^∗^	26.8 ± 2.1	2.3 (1.4–3.1)
$\dot{V}$O_2_ demand (mL⋅kg^-1^⋅min^-1^)	91 ± 7^∗^	74 ± 5	2.5 (1.6–3.4)	95 ± 9^∗^	77 ± 6	2.3 (1.4–3.1)
$\dot{V}$O_2_ demand (% of $\dot{V}$O_2max_)^#^	126 ± 14	127 ± 9	-0.1 (-0.8–0.6)	132 ± 15	131 ± 13	0.0 (-0.7–0.8)
**Gear choice and kinematics**						
G2 exclusively	*n* = 1	*n* = 9	–	–	*n* = 2	–
Speed (km⋅h^-1^)	15.0	12.9 ± 0.8	–	–	12.9 (12.7–13.2)	–
Cycle rate (Hz)	1.00	0.92 ± 0.04	–	–	1.04 (1.03–1.05)	–
Cycle length (m)	4.2	3.9 ± 0.2	–	–	–	–
G3 exclusively	*n* = 19^∗^	*n* = 3	–	*n* = 8^¤^	–	–
Speed (km⋅h^-1^)	15.9 ± 1.1	13.7 (13.5–14.2)	–	17.2 ± 1.2	–	–
Cycle rate (Hz)	0.58 ± 0.02	0.58 (0.57–0.60)	–	0.62 ± 0.03	–	–
Cycle length (m)	7.7 ± 0.6	6.7 (6.4–6.8)	–	–	–	–
G3/G2 mix	–	*n* = 2	–	*n* = 12	*n* = 12	–
Speed (km⋅h^-1^) G3						
	–	13.2 (13.1–13.4)	–	16.6 ± 1.4^G3/G2avg∗^	14.3 ± 0.9^G3/G2avg^	2.0 (1.1–2.8)
G2	–	15.1 (14.8–15.3)	–	–	–	–
Cycle rate (Hz) G3	–	0.56 (0.55–0.57)	–	0.61 ± 0.04^¤^	0.62 ± 0.03	-0.1 (-0.8–0.6)
G2	–	1.00 (1.00–1.00)	–	1.12 ± 0.09^¤^	1.07 ± 0.08	0.5 (-0.2–1.2)


*The values are presented as *mean* ±*SD* or as median and interquartile range (*n* = 14 for females and *n* = 20 for males). ES, Cohen’s *d* effect size and 95% confidence interval in parenthesis; G2, gear 2; G3, gear 3; G3/G2 mix, a mixed usage of G3 and G2; G3/G2avg, the average value for skiing with both gears. ^∗^*P* < 0.001 in comparison to females. ^¤^*P* > 0.05 in comparison to females. Statistical comparisons were performed for variables where ES is presented with exception for gear preference. ^#^Males: *n* = 19; females: *n* = 12.*

**Table 2:** Performance, power output, estimated physiological response, choice of sub-technique (i.e., gear) and cycle characteristics for the final 50 m camera section of the 1,572 m sprint time trial (CS3).

	Flat (0.3°) CS3		
	
Speed, power and metabolic demand	Males	Females	ES
Speed (km⋅h^-1^)	27.8 ± 1.5^∗^	24.4 ± 1.3	2.4 (1.5–3.2)
Total power output (W⋅kg^-1^)	6.1 ± 0.5^∗^	4.9 ± 0.4	2.3 (1.4–3.1)
Power output against gravity (W⋅kg^-1^)	0.4 ± 0.0^∗^	0.3 ± 0.0	2.4 (1.5–3.2)
Power output against snow friction (W⋅kg^-1^)	3.4 ± 0.2^∗^	3.0 ± 0.2	2.4 (1.5–3.2)
Power output against air drag (W⋅kg^-1^)	2.3 ± 0.3^∗^	1.6 ± 0.2	2.2 (1.3–3.0)
Metabolic rate (W⋅kg^-1^)	38.5 ± 3.4^∗^	31.3 ± 2.7	2.3 (1.4–3.1)
$\dot{V}$O_2_ demand (mL⋅kg^-1^⋅min^-1^)	111 ± 10^∗^	90 ± 8	2.3 (1.4–3.1)
$\dot{V}$O_2_ demand (% of $\dot{V}$O_2max_)^#^	154 ± 15	152 ± 18	0.1 (-0.7–0.8)
**Gear choice and kinematics**			
G3 exclusively	*n* = 13	*n* = 11	–
Speed (km⋅h^-1^)	27.8 ± 1.5^∗^	24.4 ± 1.2	2.5 (1.5–3.3)
Cycle rate (Hz)	0.58 ± 0.03^¤^	0.60 ± 0.03	-0.5 (-1.2–0.2)
Cycle length (m)	13.3 ± 0.7^∗^	11.4 ± 0.6	2.9 (1.9–3.8)
G4 exclusively	*n* = 3	–	–
Speed (km⋅h^-1^)	27.8 (27.7–28.8)	–	–
Cycle rate (Hz)	0.83 (0.76–0.85)	–	–
Cycle length (m)	10.0 (9.4–10.6)	–	–
G4/G3 mix	*n* = 4	*n* = 3	–
Speed (km⋅h^-1^) G4	28.8 (27.9–29.0)	26.6 (26.0–27.6)	–
G3	26.1 (25.0–27.3)	23.7 (22.7–25.2)	–
Cycle rate (Hz) G4	0.82 (0.79–0.84)	0.71 (0.71–0.72)	–
G3	0.54 (0.52–0.55)	0.61 (0.60–0.62)	–


*The values are presented as *mean* ±*SD* or as median and interquartile range (*n* = 14 for females and *n* = 20 for males). ES, Cohen’s *d* effect size and 95% confidence interval in parenthesis; G3, gear 3; G4, gear 4; G4/G3avg, a mixed usage of G4 and G3; G4/G3avg, the average value for skiing with both gears. ^∗^*P* < 0.001 in comparison to females. ^¤^*P* > 0.05 in comparison to females. Statistical comparisons were performed for variables where ES is presented. ^#^Males: *n* = 19; females: *n* = 12.*

### A Comparison of Performance and Pacing Strategy Between All Races

**FIGURE 2:**
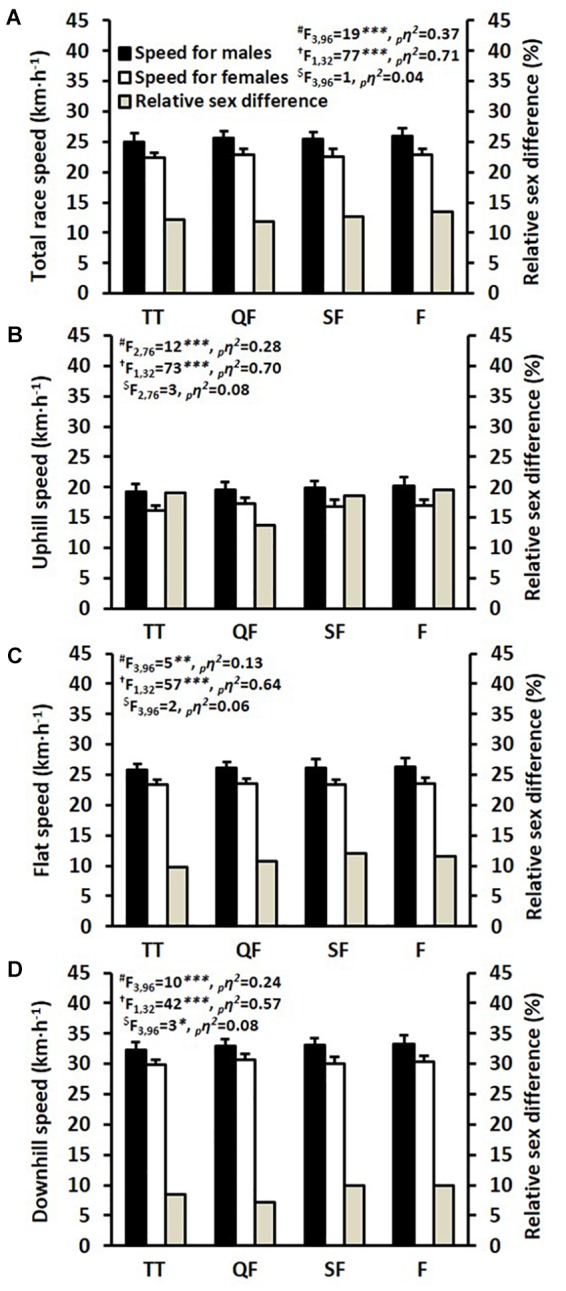
Average skiing speeds during the 1,572 m time trial (TT), quarter-final (QF), semi-final (SF), and final (F) for **(A)** the total race, **(B)** uphill terrain, **(C)** flat terrain, and **(D)** downhill terrain. ^#^Main effect for race. ^†^Main effect for sex. ^$^Interactive effect (race × sex). ^∗^*P* < 0.05, ^∗∗^*P* < 0.01, and ^∗∗∗^*P* < 0.001. $\eta_{p}^{2}$, partial eta squared effect size.

The speeds during all four races are shown in Figure 2. The peak heart rate (reached during any of the four races) was 183 ± 6 and 182 ± 7 beats⋅min^-1^ for the males and females, respectively, and this value was reached by 27, 30, 30, and 12% of the skiers in the TT, QF, SF, and F, respectively. Over the four races, the male skiers were on average 12.5% faster than the female skiers over the 1,572 m course. The most pronounced sex differences were evident on the uphill terrain, where the male skiers were on average 17.7% faster than the female skiers. By contrast, the smallest sex differences were observed on the downhill terrain, where the males were on average 8.8% faster than the females. The total race times in the QF, SF, F were 222 ± 8, 222 ± 12, and 219 ± 11 s for males and 247 ± 8, 251 ± 12, and 249 ± 11 s for females, resulting in average head-to-head race speeds that were 2.8 ± 1.7% (*P* < 0.001, ES = 1.8) and 2.4 ± 1.0% (*P* < 0.001, ES = 2.5) faster than the TT for males and females, respectively.

Mean heart rate during the TT, QF, SF, and F was 95 ± 2, 95 ± 1, 95 ± 1, and 95 ± 2% of maximum for males and 96 ± 1, 95 ± 2, 95 ± 2, and 94 ± 2% of maximum for females. There was a main effect for race (*F*_3,81_ = 5, *P* = 0.005, $\eta_{p}^{2}$ = 0.12), but not for sex (*F*_1,27_ = 0, *P* = 0.797, $\eta_{p}^{2}$ = 0.00) and there was no significant interaction effect (*F*_3,81_ = 1, *P* = 0.420, $\eta_{p}^{2}$ = 0.03). Post-race blood lactate concentration following TT, QF, SF, and F was 10.1 ± 1.9, 9.8 ± 1.4, 10.3 ± 2.0, and 11.4 ± 1.7 mmol L^-1^ for males and 9.8 ± 1.5, 8.8 ± 2.7, 9.5 ± 2.8, and 10.4 ± 3.1 mmol L^-1^ for females. Again, there was a main effect for race (*F*_3,87_ = 5, *P* = 0.002, $\eta_{p}^{2}$ = 0.16) but not for sex (*F*_1,29_ = 2, *P* = 0.190, $\eta_{p}^{2}$ = 0.06), and no interaction effect (*F*_3,87_ = 0, *P* = 0.761, $\eta_{p}^{2}$ = 0.01).

**FIGURE 3:**
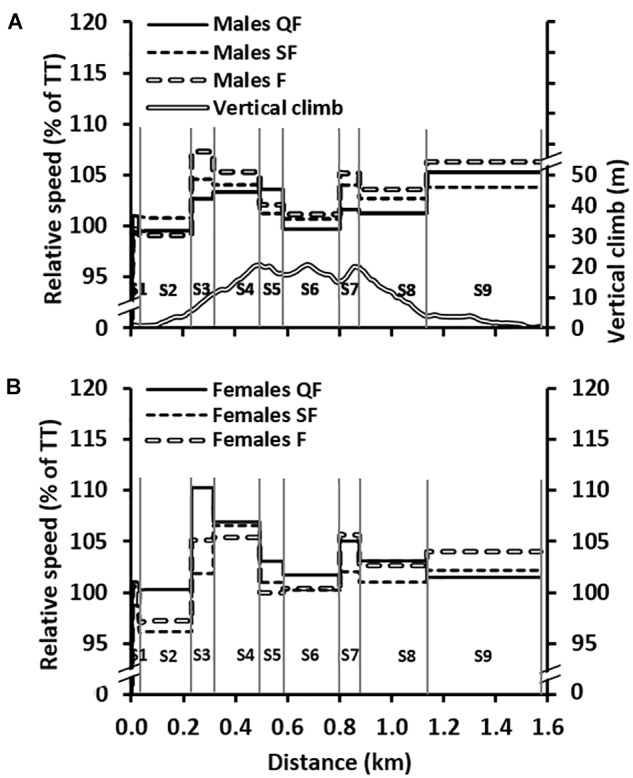
**(A)** The average section speeds relative to the time trial (TT) speeds during the head-to-head races (i.e., quarter-final [QF], semi-final [SF], and final [F]) for the males (*n* = 20) together with the course profile. **(B)** The same figure as in **(A)** but with female skiers. The vertical lines represent the nine different sections (S1–S9).

The relative differences in speeds for the head-to-head races (i.e., QF, SF, and F) versus the TT are shown in Figure 3, while mean speeds and heart rates for specific course sections (S1–S9) during the four separate sprint races are displayed in Table 3. Comparisons of the male skiers’ section speeds between the four races revealed a main effect for course section (*F*_2,46_ = 1213, *P* < 0.001, $\eta_{p}^{2}$ = 0.98) and race (*F*_3,57_ = 5, *P* = 0.003, $\eta_{p}^{2}$ = 0.22), as well as an interaction effect between course section and race (*F*_24,456_ = 8, *P* < 0.001, $\eta_{p}^{2}$ = 0.30). The same comparison for the female skiers showed a similar result (main effect for course section [*F*_3,36_ = 1838, *P* < 0.001, $\eta_{p}^{2}$ = 0.99], race [*F*_3,39_ = 12, *P* < 0.001, $\eta_{p}^{2}$ = 0.48], and an interaction effect [*F*_24,312_ = 13, *P* < 0.001, $\eta_{p}^{2}$ = 0.50]). Comparing male skiers’ heart rate values between the four races showed a main effect for section (*F*_1,25_ = 748, *P* < 0.001, $\eta_{p}^{2}$ = 0.98), with no main effect for heat (*F*_3,51_ = 1, *P* = 0.632, $\eta_{p}^{2}$ = 0.03), but an interaction effect between section and heat (*F*_24,408_ = 8, *P* < 0.001, $\eta_{p}^{2}$ = 0.33). Similar results were observed for the female skiers’ heart rate values, with a main effect for section [*F*_2,16_ = 201, *P* < 0.001, $\eta_{p}^{2}$ = 0.95], no main effect for heat [*F*_3,30_ = 2, *P* = 0.149, $\eta_{p}^{2}$ = 0.16], but an interaction effect between section and heat [*F*_24,240_ = 4, *P* < 0.001, $\eta_{p}^{2}$ = 0.30]).

**Table 3:** Mean ± SD speeds and heart rates (HR) in specific sections (S1–S9) for the four separate sprint races (i.e., the time trial [TT], quarter-final [QF], semi-final [SF], and final [F]) together with the tactical positioning during the head-to-head (HH) races (i.e., QF, SF, and F).

		Course sections (S1–S9)								
		
Race	Speed (km⋅h^-1^)	S1 (-0.5°)	S2 (1.4°)	S3 (3.9°)	S4 (3.1°)	S5 (-1.9°)	S6 (-0.7°)	S7 (3.7°)	S8 (-3.6°)	S9 (-0.5°)
TT	Males	23.2 ± 0.7	25.7 ± 1.7	18.3 ± 1.4	18.4 ± 1.3	25.6 ± 1.4	23.6 ± 0.8	23.0 ± 1.5	35.5 ± 1.1	27.2 ± 1.2
	Females	21.2 ± 0.7	22.6 ± 1.0	15.3 ± 0.9	15.3 ± 1.0	22.9 ± 0.8	21.7 ± 0.7	19.7 ± 1.1	33.2 ± 1.1	25.0 ± 0.9
QF	Males	23.4 ± 0.8	25.5 ± 0.9	18.7 ± 1.6	18.9 ± 1.1	26.4 ± 1.9	23.5 ± 0.7	23.3 ± 1.4	36.0 ± 1.7	28.7 ± 1.8
	Females	21.4 ± 0.7	22.7 ± 0.5	16.9 ± 0.8	16.4 ± 1.0	23.6 ± 0.9	22.0 ± 0.7	20.7 ± 1.4	34.3 ± 1.0	25.4 ± 1.1
SF	Males	23.0 ± 1.2	25.8 ± 1.0	19.0 ± 1.1	19.0 ± 1.2	25.9 ± 1.4	23.8 ± 1.1	23.9 ± 2.0	36.5 ± 1.8	28.3 ± 2.6
	Females	20.9 ± 1.0	21.7 ± 0.8	15.5 ± 0.6	16.4 ± 1.5	23.1 ± 1.2	21.7 ± 1.1	20.1 ± 1.6	33.6 ± 1.4	25.6 ± 1.2
F	Males	23.1 ± 0.9	25.4 ± 1.5	19.6 ± 1.2	19.3 ± 1.4	26.1 ± 1.0	23.9 ± 1.2	24.1 ± 1.9	36.8 ± 1.8	28.9 ± 1.9
	Females	21.3 ± 1.3	22.0 ± 0.8	16.1 ± 0.7	16.1 ± 1.0	22.9 ± 1.1	21.7 ± 0.9	20.8 ± 1.9	34.1 ± 1.4	26.0 ± 1.3
**Race**	**HR (% of maximum)^#^**									
TT	Males	70 ± 4	88 ± 4	95 ± 2	97 ± 2	98 ± 1	98 ± 1	98 ± 1	97 ± 1	97 ± 1
	Females	71 ± 7	90 ± 4	97 ± 2	98 ± 1	99 ± 1	98 ± 1	97 ± 2	97 ± 2	97 ± 2
QF	Males	69 ± 5	89 ± 2	95 ± 1	97 ± 1	97 ± 1	97 ± 1	97 ± 1	97 ± 1	98 ± 1
	Females	69 ± 5	90 ± 3	96 ± 2	97 ± 1	98 ± 2	97 ± 2	97 ± 2	96 ± 3	96 ± 2
SF	Males	72 ± 4	89 ± 2	95 ± 1	96 ± 1	97 ± 1	97 ± 1	97 ± 1	96 ± 1	97 ± 1
	Females	72 ± 5	90 ± 3	95 ± 2	97 ± 1	97 ± 2	96 ± 2	96 ± 2	95 ± 3	96 ± 2
F	Males	74 ± 5	89 ± 3	95 ± 2	96 ± 2	97 ± 2	97 ± 2	96 ± 2	96 ± 2	97 ± 2
	Females	73 ± 6	90 ± 3	95 ± 1	96 ± 1	96 ± 2	96 ± 2	96 ± 2	95 ± 3	96 ± 2
**HH races**	**Correlation with S9^†^**									
	Kendalls tau *b*	0.51	0.63	0.66	0.72	0.73	0.75	0.78	0.84	–
	95% CI	0.37–0.64	0.50–0.77	0.53–0.79	0.61–0.83	0.62–0.84	0.64–0.86	0.67–0.89	0.77–0.92	–
**HH races**	**The winner’s position^†^**									
	1st	61%	61%	67%	67%	67%	67%	61%	72%	–
	2nd	22%	28%	28%	22%	22%	22%	28%	22%	–
	3rd	11%	6%	0%	6%	6%	6%	6%	6%	–
	4th	6%	6%	6%	6%	6%	6%	6%	0%	–
	≥5th	0%	0%	0%	0%	0%	0%	0%	0%	–


*CI, confidence interval. ^#^Males: *n* = 18; females: *n* = 11. **^†^**The “Correlation with S9” data are the correlations between positioning and final rank, while “The winner’s position” data are the relative frequencies (in %) of all the race winners’ positions at the end of each section. These data are based on all 18 HH races (six for females [*n* = 7 per race] and 12 for males [*n* = 5 per race]).*

Correlations between course section rankings and final rankings based on all 18 head-to-head races (i.e., all QF, SF, and F races) are also shown in Table 3. The average correlation coefficients ranged from 0.51 to 0.84, with a gradual increase from S1 to S8. In addition, descriptive data showing the relative frequencies of all head-to-head race winners’ positions at the end of each section are displayed in Table 3 and show that approximately two-thirds of head-to-head race winners were positioned at the front of the race already after the first section.

## Discussion

This investigation of differences in performance and pacing strategies between sexes during a sprint skiing competition has shown:

(1) An overall sex difference in TT performance of 12%, with male skiers considerably (19%) faster on the uphill sections, where they also used G3 more frequently than the females;
(2) That the speed profile and power output distribution along the course were highly variable for both sexes, while the variation in heart rate was low;
(3) That the head-to-head races (i.e., QF, SF, and F) were 2.6 and 2.1% faster than the TT for the male and female skiers, respectively, with main differences between the head-to-head races and the TT observed during the uphill and final flat sections.

### Sex Differences in TT Performance

As hypothesized, the male skiers were considerably faster than the females during the TT, by a magnitude of ∼12%, which has also been observed for middle- and long-distance running (Coast et al., 2004; Cheuvront et al., 2005). Unlike studies performed in the laboratory, the influence of air drag on cross-country skiing performed outdoors is an important factor to consider. For instance, during the TT in the current study, the males generated an 18.2% higher relative power output than the females, while the difference in speed was considerably lower at 12.2%. However, if a similar TT was conducted on a treadmill, differences in relative power output and speed had been similar due to the negligible air drag (Andersson et al., 2017). Hence, the influence of air drag clearly reduces outdoor performance differences between athletes of differing capacities and body sizes, with this effect amplified at increased average speeds (di Prampero, 1986; Bergh, 1987; Jeukendrup et al., 2000). In the current study, the greatest difference in speed between sexes was observed during uphill skiing (being 19% higher for the males), where power exerted against air drag constitutes a minor part of the total power output. Higher uphill speeds among male cross-country skiers have been observed previously by Sandbakk et al. (2012), who demonstrated 17% higher peak speeds for males compared with females during an indoor incremental time-to-exhaustion test using G3 at a 2.9° incline. This appears to be related to a divergence between the sexes as the relative contribution from the upper body increases (Sandbakk et al., 2014), with a greater relative reliance on the upper body for propulsion during ski-skating on uphill compared to level and downhill terrain (Millet et al., 1998; Smith, 2003).

For endurance sports in general, performance differences between sexes have been attributed to differences in $\dot{V}$O_2max_, where females typically display ∼10–15% lower relative values, which are assumed to be mainly due to a lower relative hemoglobin mass in females (Cheuvront et al., 2005; Sandbakk et al., 2012). In the current study, the female skiers had a 17.8% lower relative $\dot{V}$O_2max_. Although oxygen uptake was not measured during the sprint competition, no difference in relative heart rate was observed between the sexes, which indicates a similar fractional utilization of $\dot{V}$O_2max_ (Londeree and Ames, 1976) between the male and female skiers. Moreover, even though blood lactate concentration is only an indirect marker of anaerobic energy production, the similar post-race blood lactate concentrations between sexes indicate a comparable anaerobic energy production (Vandewalle et al., 1987). Thus, it is likely that a major part of the observed sex difference in sprint skiing performance was related to differences in $\dot{V}$O_2max_.

In ski-skating, gear choice is likely to be most prominently influenced by a combination of factors such as speed, slope incline, technical skill and physiological ability (Kvamme et al., 2005; Andersson et al., 2010; Losnegard et al., 2012). Previous findings by Andersson et al. (2010) showed that faster skiers use higher gears during uphill skiing, which is also supported by findings from the current study, whereby most of the faster males used G3 exclusively and most of the females used G2 exclusively. In addition, the fastest female skier over the first camera section used G3 exclusively and was 10% faster than the female group average. Although the different gear choices between males and females made the comparison of gear-specific cycle characteristics problematic, these data indicate that male skiers generate higher speeds in G3 compared to the females using G3, primarily due to longer cycle lengths at relatively similar cycle rates (see Tables 1, 2).

### Pacing Strategy and Performance During the TT

To our knowledge, this is the first study to analyze pacing strategies during a full sprint skiing competition on snow. Although pacing is usually described as being positive, even or negative, the variable terrain in cross-country skiing, together with the fact that skiers typically race only one lap on a given course, make such classifications inappropriate. In order to best simulate World Cup conditions, the current study was conducted on a one-lap course over varying terrain. As a result, the skiers employed a variable pacing strategy in terms of power output estimated from the TT, resulting in a highly variable metabolic requirement over the course. The estimated metabolic demands for the two uphill camera sections (i.e., ∼76 mL kg^-1^ min^-1^ for the females and ∼93 mL kg^-1^ min^-1^ for the males) were ∼129%

of $\dot{V}$O_2max_ for both sexes, indicating a considerable anaerobic energy yield during uphill skiing. Supramaximal intensities for uphill roller-skiing using the skating technique have recently been observed during a 15-km outdoor TT race, with reported uphill exercise intensities of ∼115% of $\dot{V}$O_2max_ for relatively short (∼65 m) and steep (8.7°) climbs preceded by a relatively flat section (Karlsson et al., 2018). Hence, a skier’s ability to generate and tolerate uphill exercise intensities above $\dot{V}$O_2max_ is likely to be an important factor for predicting both sprint and distance cross-country skiing performance. Interestingly, as shown in Figure 1B, the heart rate was relatively stable from the beginning of the fourth section until the finish. Since heart rate can be viewed as a surrogate marker of relative oxygen uptake (i.e., percent of $\dot{V}$O_2max_) (Londeree and Ames, 1976), it is likely that most of the variation in power output, and hence metabolic requirement, was due to a fluctuating anaerobic energy yield along the course, as has been previously shown for classic roller-skiing (Andersson et al., 2016, 2017). For sprint cross-country skiing modeled on an undulating course, Sundström et al. (2013) predicted improved performance with a more variable power output profile (i.e., higher power output on uphill terrain) in order to achieve a more even speed profile, thereby reducing air drag (van Ingen Schenau et al., 1992). Therefore, if a skier improves his/her ability to repeatedly generate and tolerate high levels of anaerobic energy production on uphills, with efficient recovery on downhills at an elevated $\dot{V}$O_2_ in comparison to the $\dot{V}$O_2_ demand, the utilization of aerobic and anaerobic metabolic resources may be enhanced. This would also likely improve the pacing strategy from a mechanical perspective, and thereby performance, due to a more even speed profile.

For the relative speed profile, expressed as a percentage of the average TT speed, the between-athlete coefficient of variation across the course sections was relatively small (2–4%), indicating relatively similar pacing strategies within each sex group. However, an interaction effect between the sexes was observed for the relative speed profile (Figure 1C), where females demonstrated a higher variation in relative speed. This was mainly due to relatively slower speeds on the uphills and faster speeds on the downhills, in comparison to the males. Since the power output required to overcome air drag increases exponentially with skiing speed, it is likely that faster skiers, in general, would demonstrate a less variable speed profile than that of slower skiers (i.e., that the more even speed profile for faster than slower skiers is an outcome rather than the aim, *per se*). That is because the power output required to overcome air drag is the cube of the skiing speed; hence, at relatively high speeds where a major part of the power output is used to overcome air drag, a small increase in speed requires an exponential increase in the metabolic demand (di Prampero, 1986; Jeukendrup et al., 2000). This is exemplified when comparing the relative differences in speed and metabolic rate between the sexes for the three camera sections. Of note, the power output and metabolic rate were both 23% higher for males than females on the two uphill camera sections and the flat camera section, while the difference in speed was 20% for uphill skiing versus 14% on the flat camera section. This may explain why differences in speeds between sexes were lower for flat and downhill terrain than for uphill terrain in the current study, and why overall sex differences in performance are lower for “high-speed” events such as speed skating and cycling (∼7.9%) than were observed in the current study (12.2%) (Thibault et al., 2010). Therefore, differences in TT performance over different sections of the course for the males and females in the current study were probably due to a combined effect of differences in the total metabolic resources, mainly due to differences in $\dot{V}$O_2max_, together with an external air drag effect on the relative speed profile.

### Head-to-Head Sprint Skiing Performance, Pacing Strategy, and Race Tactics

A unique characteristic of sprint cross-country ski competitions is that athletes complete four separate races on the same day, where the TT qualification race (i.e., the prolog) is without direct contact with opponents and the remaining three knockout races involve six skiers racing head-to-head. While the aim of the TT prolog is to ensure qualification to the QF (i.e., a final ranking in the top 30), the focus of the head-to-head races is to finish in the top two (for the QF and SF) or first (in the F). Speed is therefore of less importance during the head-to-head races and pacing is likely to be influenced by other competitors’ race tactics. However, this dynamic is made more complex by the fact that the two fastest skiers in each knockout round who finish outside of the top two in each heat qualify to the next round as “lucky losers,” making final race time not entirely redundant. In the current study, the three head-to-head races were on average 2.6 and 2.1% faster than the TT for the males and females, respectively. Although not all skiers would have benefited similarly from drafting, particularly the race winners who were typically leading after the first 30 m, the improved average performances in the head-to-head races were probably partly due to the reduced air-drag resulting from drafting. The presence of direct competitors has also been shown to affect performance, with cycle TT performance improving by ∼2.5% when moderately-trained individuals raced against a virtual opponent compared to no opponent (Corbett et al., 2012; Konings et al., 2017). However, the elite athletes in the current study would be expected to have a high internal motivation, irrespective of whether they were racing head-to-head or alone (Corbett et al., 2012). Thus, the effects of external stimuli (i.e., direct contact with opponents) could be lower for the current group of participants compared with lower-level athletes or non-athletes.

Even though main effects of race (i.e., TT, QF, SF, and F) were observed for heart rate and blood lactate concentration, differences in these physiological responses were relatively small between the four races. Therefore, it is likely that the advantage of drafting other skiers would be the main contributing factor for the faster speeds achieved during the head-to-head races, rather than greater effort. As shown in Figure 3, the speed profiles during the head-to-head races differed compared to the TT, with higher speeds achieved on the main uphill sections (S3, S4, and S7) and for the final section (S9). This was possibly a direct cause of breakaway attempts by skiers on the uphill sections, due to the relatively lower benefit of drafting when speed is lower (Bilodeau et al., 1994; Brisswalter and Hausswirth, 2008). The relatively higher speeds observed over S9 was likely due to faster end spurts in the head-to-head races since these races are determined by final position rather than total race time.

The current study is the first to our knowledge to have analyzed the associations between positioning and performance in head-to-head knockout races in sprint skiing. As shown in Table 3, most of the head-to-head race winners were already leading after the first section (i.e., after 30 m), and the correlation coefficients between positioning on the course and final rank gradually increased with increased distance. The moderate correlations at the early stages of the race in cross-country sprint skiing are in contrast to sports such as 1500-m short-track speed-skating and sprint track cycling, where most race winners draft behind other competitors during the early stages of the race (Moffatt et al., 2014; Konings et al., 2016). This difference is most likely related to the lower absolute speeds and the substantially lower air frictional losses in sprint skiing, which lead to a lower relative advantage of drafting. However, the energy saving benefits of drafting in cross-country skiing should not be underestimated, especially for fast skiing over flat and downhill terrain. For instance, drafting another skier (of a similar performance level) at an average speed of 20.1 km h^-1^ lowered relative heart rate by five percentage points during 2 km of high-intensity ski-skating compared to the leading skier (Bilodeau et al., 1994). As shown in Figure 3, the knockout races were finished with a more pronounced end spurt than the TT, making rapid anaerobic energy supply at the very end of these races potentially decisive for successful performance. Therefore, a skier’s maximal speed capacity and the ability for rapid accelerations are probably more important in knockout sprint races, where head-to-head positioning exerts a direct impact on the race result.

### Limitations

The results presented in the current study are derived from a relatively large number of male and female skiers with good control and organization for this type of applied field study. However, it is acknowledged that the simulation was not entirely representative of a World Cup competition, since all skiers completed all four races and were not knocked out through the rounds. This method was adopted for the obvious reason of maintaining a participant group of *n* = 34 throughout the entire study. As a result, performance was defined as total race time, which differs from a real-world scenario where final positions in the head-to-head races would determine further qualification and performance.

It is also acknowledged that data presented for power output and metabolic demand were based on some assumptions. For instance, the friction coefficient, effective drag area and gross efficiency for the calculations of power output and metabolic demand were estimated based on previous studies and additional unpublished data. Also, gross efficiency determined for roller-skiing may not be exactly the same as for on-snow skiing in slow snow conditions (i.e., wet, fresh snow). From the glide-test results, it can be noted that the gliding properties of the skis were slightly altered from pre- to post-competition, with downhill speeds ∼1% slower following the competition. Moreover, the power output estimates were solely based on average section speeds and the influence of instantaneous changes of momentum on the power output estimate was neglected. The accuracy of the propulsive power output and metabolic demand measures could, therefore, be improved by employing more sophisticated individual assessments of these parameters. Thus, future studies may benefit from the use of high-accuracy GNSS equipment, which would enable detailed analyses of a skier’s position and speed along the course, preferably also combined with a portable respiratory gas analyzer for the quantification of oxygen uptake.

## Conclusion and Perspectives

The present findings reveal an overall sex difference in sprint cross-country skiing performance of ∼12.5%. This difference in performance was significantly affected by terrain type, where considerably larger sex differences were observed during uphill compared with downhill and flat skiing. Moreover, the females were 19% slower uphill than the males during the TT, with females using more of G2 compared to the males who mainly used G3. The female skiers also demonstrated a more variable speed profile than the males.

The power output distribution along the course was highly variable, with the highest power outputs being generated on the uphill sections, while the variation in heart rate was low. This suggests a highly variable anaerobic energy yield along the course. The head-to-head races were on average 2.4% faster than the TT for both sex groups, while average heart rate and post-race blood lactate concentrations were relatively similar for all races. Therefore, the faster race times during the head-to-head races may be primarily related to reduced air-drag due to drafting.

The results presented in the current study provide important insight into the sex differences associated with performance and pacing strategies during field-based sprint skiing on snow in a group of elite cross-country skiers. A novel finding was that the relative sex difference in estimated relative power output for uphill and flat skiing during the TT was similar, while the associated relative difference in speed was substantially higher for uphill than flat skiing. This differs from laboratory studies using treadmill roller-skiing, where differences in relative power output and speed between sexes are similar due to negligible air-drag (Sandbakk et al., 2012, 2014). This highlights the importance of conducting studies of these elite athletes in field-based environments. An additional finding was the more variable power output distribution along the course compared with the heart rate response, which indicates an intermittent need for anaerobic energy production. More importantly, from an applied perspective this finding confirms that heart rate conveys an incomplete picture for describing exercise intensity during sprint skiing, which has been suggested previously for other modes of exercise (Buchheit and Laursen, 2013).

## Data Availability

The datasets generated for this study are available on request to the corresponding author.

## Author Contributions

EA performed the data and statistical analysis, interpreted the results and wrote the first draft. EA, AG, OS, and KM designed the study, collected the data, revised the manuscript, approved the final version, and agreed to be accountable for all aspects of the work.

## Conflict of Interest Statement

The authors declare that the research was conducted in the absence of any commercial or financial relationships that could be construed as a potential conflict of interest.

## Footnotes

1 www.kinovea.org
